# Combined posterolateral knee reconstruction: ACL-based injuries perform better compared to PCL-based injuries

**DOI:** 10.1007/s00167-020-06409-3

**Published:** 2021-01-23

**Authors:** Patricia M. Lutz, Michael Merkle, Philipp W. Winkler, Stephanie Geyer, Elmar Herbst, Sepp Braun, Andreas B. Imhoff, Matthias J. Feucht

**Affiliations:** 1 Department for Orthopedic Sports Medicine, Technical University Munich, Ismaninger Str. 22, 81675 Munich, Germany; 2 Department of Trauma, Hand and Reconstructive Surgery, University Hospital Muenster, Westfalian-Wilhelms University Muenster, Muenster, Germany; 3 Department of Orthopedics and Trauma Surgery, Medical Center, Faculty of Medicine, Albert-Ludwigs-University of Freiburg, Freiburg, Germany; 4 Gelenkpunkt - Sports- and Traumasurgery Innsbruck, Innsbruck, Austria; 5 Research Unit for Orthopaedic Sports Medicine and Injury Prevention, Private University for Health Sciences, Medical Informatics and Technology, Hall, Austria

**Keywords:** Posterolateral corner, Posterior cruciate ligament, Anterior cruciate ligament, Return to sport, Physical activity, Return to work, Complex ligamentous knee injury, Multiligament knee injury

## Abstract

**Purpose:**

To compare post-operative physical activity and return to work after combined posterolateral corner (PLC) reconstruction (PLC-R) in anterior cruciate ligament (ACL)- or posterior cruciate ligament (PCL)-based injuries.

**Methods:**

Patients aged > 18 years undergoing PLC-R using the Larson technique combined with either ACL or PCL reconstruction were included. Outcome was evaluated retrospectively after a minimum follow-up of 24 months using Tegner Activity Scale, Activity Rating Scale (ARS), Knee Injury and Osteoarthritis Outcome Score (KOOS), work intensity according to REFA classification, and a questionnaire about type of occupation and time to return to work.

**Results:**

A total of 32 patients (11 ACL-based injuries and 21 PCL-based injuries) were included. Mean follow-up was 56 ± 26 months in the ACL-based injury group and 59 ± 24 months in the PCL-based injury group. All patients in the ACL-based injury group and 91% of patients in the PCL-based injury group returned to sports activities. Comparing pre- and post-operative values, a significant deterioration of the Tegner Activity Scale and ARS was observed in the PCL-based injury group, whereas no significant change was observed in the ACL-based injury group. KOOS subscales were generally higher in the ACL-based injury with significant differences in the subscale sports and recreational activities. Patients with ACL-based injuries returned to work significantly earlier compared to patients with PCL-based injuries (11 ± 4 weeks vs. 21 ± 10 weeks, *p* < 0.05).

**Conclusion:**

High rates of return to sports and work can be expected after combined PLC-R in both ACL- and PCL-based injuries. However, deterioration of sports ability must be expected in PCL-based injuries. ACL-based injuries led to superior patient-reported outcomes and an earlier return to work, as compared to PCL-based injuries.

**Level of evidence:**

Level IV.

## Introduction

Posterolateral corner (PLC) injuries of the knee are relatively rare and most commonly combined with concomitant ligamentous injuries [5, 7, 21]. Injuries to the PLC result in functional impairment, involving instability and pain which consecutively may accelerate the development of knee osteoarthritis [16].

Posterior cruciate ligament (PCL) tears are frequently combined with injuries to the PLC [10, 21, 30, 32, 42]. Biomechanically, isolated PCL reconstruction (PCL-R) is not able to restore native knee kinematics in combined PCL-PLC injuries [13, 24, 36]. However, while many authors focused on combined PCL-PLC injuries, little is known about less common combined anterior cruciate ligament (ACL) and PLC injuries [18, 21, 37, 39]. From a biomechanical point of view, insufficiency of the PLC increases varus load on the ACL graft, which may lead to a higher failure rate after isolated ACL reconstruction (ACL-R) [20, 29, 45]. Therefore, ACL-R or PCL-R should be combined with PLC-R in combined injuries to restore nearly normal biomechanics [6, 33].

Satisfying functional outcomes have been reported after PLC-R combined with either ACL-R [2, 34, 43] or PCL-R [9, 11, 17, 25, 40]. However, these usually young and active patients have high demands concerning post-operative sports activity and work ability. These specific outcome measures are still underreported in the current literature. With our study, an improved patient education regarding post-operative sports activity, work ability, and return-to-work time following such complex ligamentous knee injuries can be achieved.

The purpose of this study was to compare post-operative sports activity and work ability between patients undergoing combined PLC-R for either ACL- or PCL-based injuries using validated patient-reported outcome scores.

The hypothesis was that PCL-based PLC injuries would result in lower return-to-sports rates and reduced work ability as compared to ACL-based injuries.

## Materials and methods

This retrospective study was conducted to compare post-operative sports activity and work ability in patients undergoing combined PLC-R for either ACL- or PCL-based injuries. The study was approved by the institutional review board of the Technical University of Munich (520/17 S) and conducted according to the Declaration of Helsinki. All subjects gave their written informed consent to participate in this investigation.

For the purpose of the study, patients undergoing combined PLC-R with either ACL-R or PCL-R between 2011 and 2017 were included. Indications for PLC-R were: acute or chronic grade III PLC injury confirmed by (1) magnetic resonance imaging and (2) clinical examination (positive varus stress test at 0° and 30° of knee flexion and positive dial test at 30° of knee flexion). Further inclusion criteria were: age > 18 years and post-operative follow-up of at least 24 months. Exclusion criteria were: relevant comorbidities (infectious diseases, cancer, severe cardiovascular diseases), previous ligamentous reconstructions to either knee, concomitant osteotomy, medial collateral ligament reconstruction, lack of language skills, and missing consent to participate.

### Operative technique and post-operative rehabilitation

In both groups, an isometric reconstruction of the PLC based on the description of *Larson* with a semitendinosus tendon autograft was performed [22]. Briefly, a 4–5-mm bone tunnel was created in the fibular head in an anterolateral to posteromedial direction. For the femoral tunnel, a K-wire was placed anterior and cranial to the lateral epicondyle. After isometry testing, the guidewire was overreamed with a cannulated drill according to the diameter of the graft. Subsequently, the graft was passed through the fibular tunnel and fixed with a bio-absorbable tenodesis screw (Arthrex, Naples, USA). The graft was then passed underneath the biceps tendon and iliotibial band and pulled inside the femoral tunnel. Femoral fixation was done in 30° knee flexion, slight internal rotation, and valgus stress with a bio-absorbable interference screw (Arthrex, Naples, USA).

In the ACL-based injury group, an anatomic ACL-R technique with hamstring tendon autografts was performed. The femoral tunnel was drilled via an anteromedial portal according to the diameter of the graft. The tibial tunnel was placed in the centre of the tibial ACL footprint. The graft was secured at 20° of flexion with an extra-cortical suspension device (ACL TightRope, Arthrex, Naples, USA) on the femoral site and with a bio-absorbable interference screw (Arthrex, Naples, USA) tibially.

In the PCL-based injury group, an anatomic single-bundle PCL-R technique (anterolateral bundle reconstruction) with a hamstring or quadriceps tendon autograft was performed. The femoral tunnel was drilled via a deep anterolateral portal according to the diameter of the graft. A guidewire was then placed in the centre of the tibial PCL footprint and overreamed according to the diameter of the graft. After graft passage, fixation was performed using bio-absorbable interference screws (Arthrex, Naples, USA).

The post-operative rehabilitation protocol in the ACL-based injury group consisted of 6 weeks of partial weight bearing on crutches. During the first two weeks, a hinged brace (Medi M4, Medi Bayreuth, Germany) with limited range of motion (ROM) (extension/flexion 0°/20°/90°) was used. After 2 weeks, ROM was limited to extension/flexion 0°/10°/90° for 4 weeks.

The post-operative protocol in the PCL-based injury group consisted of 6 weeks of partial weight bearing on crutches. During the first 6 weeks, a specific brace in full extension with posterior tibial support (Medi PTS, Medi Bayreuth, Germany) was used. Only passive ROM of 0°/0°/90° in prone position was allowed. After 6 weeks, a hinged brace with posterior tibial support (Medi PCL Dynamic or Medi PCL Jack, Medi Bayreuth, Germany) was used without limitations in ROM for additional 18 weeks.

Return-to-sport-specific training in both groups was allowed after 6 months and full return to contact and/or pivoting sports activities after a minimum of 9 months post-operatively.

### Data collection

Medical records were reviewed to collect patient demographics and details about the medical history and surgery.

Physical activity was evaluated by Tegner Activity Scale [38], Activity rating scale [28], and a questionnaire regarding sports disciplines, sports ability, and frequency. Knee Injury and Osteoarthritis Outcome Score (KOOS) was used to evaluate self-administered knee function [35]. Time to return to work was calculated. Furthermore, work ability was evaluated by the use of questions regarding pre- and post-operative type of occupation and work intensity (according to “REFA” classification) [15].

### Statistical analysis

Statistical analysis was performed using SPSS software version 25.0 (IBM-SPSS, New York, USA). Continuous variables were calculated as mean ± standard deviation allowing one decimal. Categorical variables were reported as count and percentages allowing no decimal. Normal distribution of all data was evaluated with the Shapiro–Wilk test. The Wilcoxon signed-rank test was used to compare preoperative and post-operative nonparametric continuous variables allowing three decimals. Group comparison for continuous variables was performed with Mann–Whitney *U* test and unpaired *t* test, as appropriate, allowing three decimals. The chi-square test was used to compare categorical variables allowing three decimals. A *p* value of less than 0.05 was considered to indicate statistical significance.

Post-operative sports activity was considered to be the primary outcome measure. Accordingly, the KOOS subscale sports and recreational activities were used for a priori power analysis. Assuming a standard deviation of 20 points and a mean value of 50 and 72 points for combined ACL- and PCL-based PLC injuries, respectively, an effect size of 1.1 was calculated [41]. Considering the lower incidence of ACL-based PLC injuries compared to PCL-based PLC injuries, a group allocation of 1:2 was assumed. Consequently, a total sample size of 32 patients (ACL-based PLC injuries, *n* = 11; PCL-based PLC injuries, *n* = 21) was required to achieve a statistical power of 0.8.

## Results

**Fig. 1:**
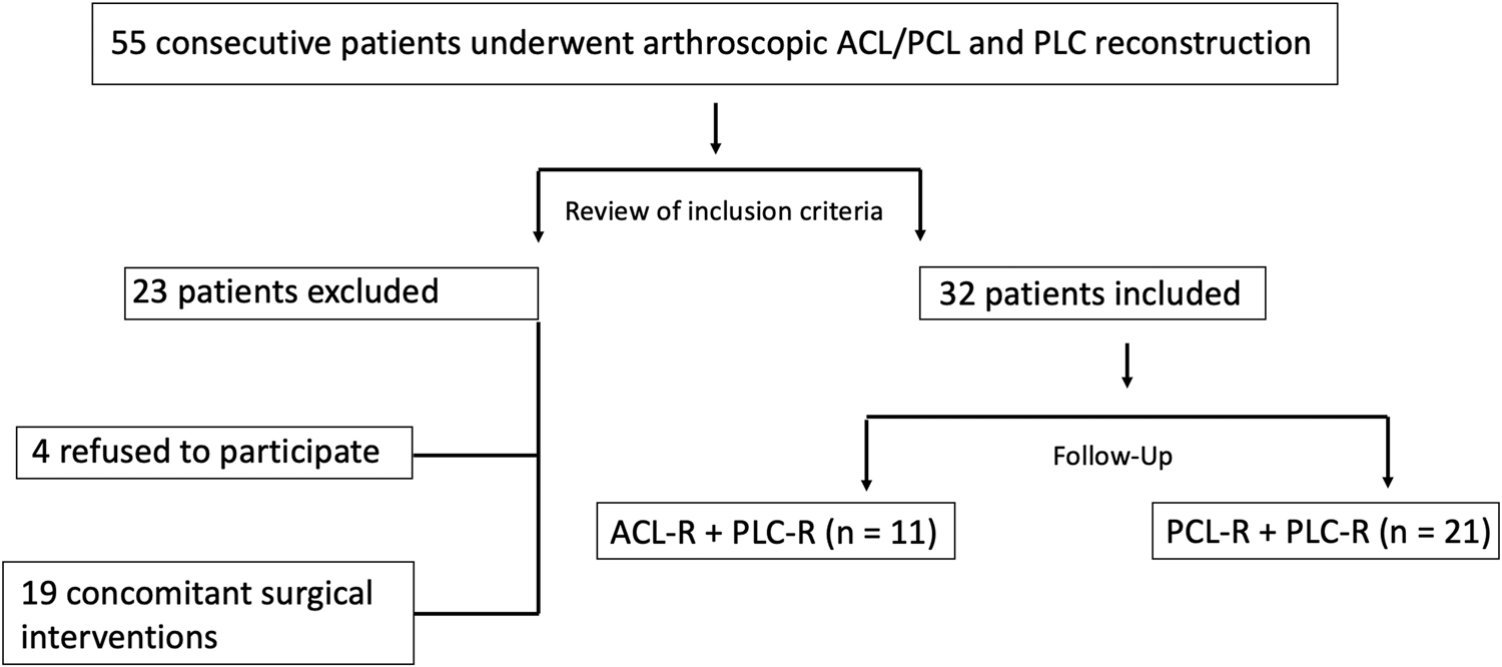
Flow chart of patient enrolment. Concomitant surgical interventions that were excluded were: ACL + PCL and PLC reconstruction (*n* = 8), MPFL reconstruction (*n* = 2), MCL reconstruction or repair (*n* = 9); *ACL* anterior cruciate ligament, *MCL* medial collateral ligament, *MPFL* medial patellofemoral ligament, *PCL* posterior cruciate ligament, *PLC* posterolateral corner, *R* reconstruction

Out of 36 patients who met the inclusion criteria, a total of 32 patients could be included for final analysis (follow-up rate, 89%). Details of enrolment are shown in Fig. 1.

Demographic data are shown in Table 1. No significant differences with regard to demographics were observed between the two groups.

**Table 1:** Descriptive statistics of the demographic data and main parameters of the total study group

	ACL-based injury	PCL-based injury	*p* value
Number of patients, *n*	11	21	
Follow-up (months)	55.7 ± 25.5 (25–94)	59.0 ± 24.2 (24–101)	n.s
Age at surgery (years)	30.0 ± 6.1 (22–41)	34.0 ± 14.0 (18–63)	n.s
Sex, *n* (%)			n.s
Male	10 (91%)	17 (81%)	
Female	1 (9%)	4 (19%)	
BMI (kg/m^2^)	25.2 ± 2.3 (22–29)	25.5 ± 3.9 (19–32)	n.s
Time to surgery (months)	23.3 ± 52.0 (0–174)	33.2 ± 49.4 (1–181)	n.s
Concomitant procedures, *n* (%)			
None	9 (82%)	14 (67%)	
Partial resection of meniscus	2 (18%)	5 (24%)	
Meniscus repair	0 (0%)	2 (10%)	
Post-operative complications, *n* (%)			
None	11 (100%)	19 (90%)	
Infection with Staphylococcus epidermidis	0 (0%)	1 (5%)	
Re-instability	0 (0%)	1 (5%)	

Continuous variables are shown as mean ± standard deviation (range); categorical variables are shown as percentages

*ACL* anterior cruciate ligament, *BMI* body mass index, *n.s.* not significant, *PCL* posterior cruciate ligament, *PLC* posterolateral corner

**Fig. 2:**
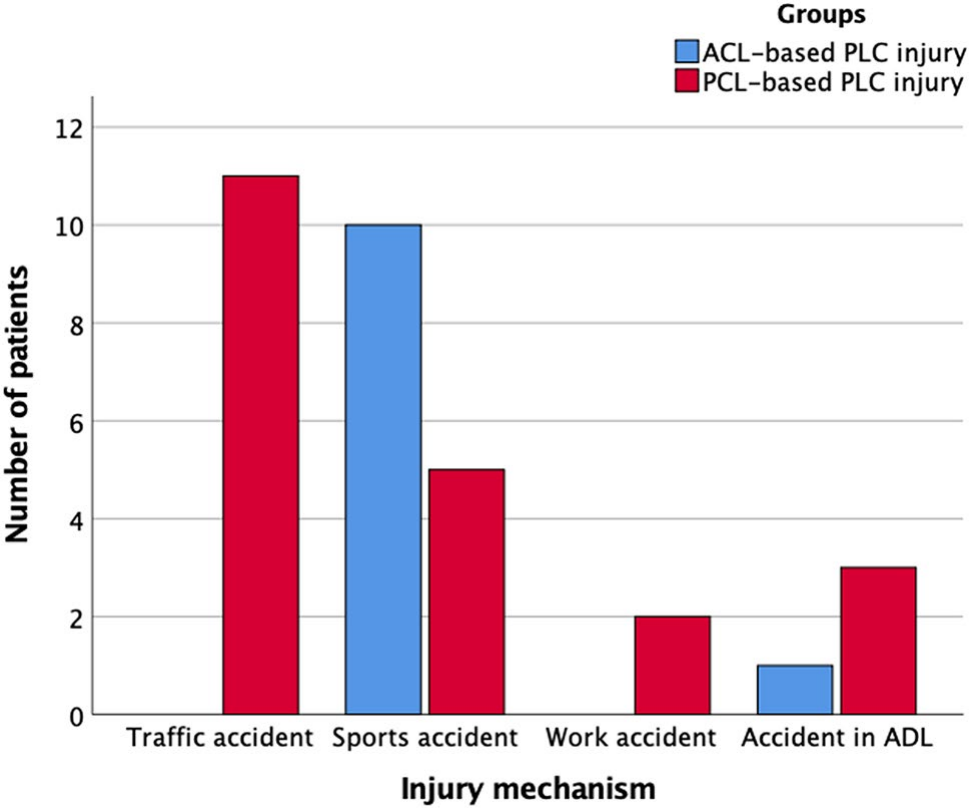
PLC injury mechanism in ACL-based injuries and PCL- based injuries. Group comparison revealed a statistically significant difference with respect to the injury mechanism (*p* < 0.001) *ADL* activities of daily living, *PLC* posterolateral corner, *ACL* anterior cruciate ligament, *PCL* posterior cruciate ligament

The most common cause of ACL-based injuries was sports injuries (82%), followed by accidents during activities of daily living (18%). The most common cause of PCL-based injuries was traffic accidents (52%), followed by sports injuries (24%), and accidents during activities of daily living (14%) or work (10%) (Fig. 2). Statistically significant more sport-related injuries were observed in the ACL-based injury group as compared to the PCL-based injury group (82% vs. 24%, *p* < 0.001). On the other hand, traffic-related injuries were more frequent in the PCL-based injury group (52% vs. 0%, *p* = 0.003).

### Sports activity

Results of sports activity are summarized in Table 2.

**Table 2:** Physical activity and clinical outcome scores of the total study group

	ACL-based injury	PCL-based injury	p value
TEGNER post-operative	5* (2–8)	4* (1–8)^$^	n.s
TEGNER preoperative	6* (2–10)	5* (3–10)^$^	n.s
ARS post-operative	3.0 ± 3.5 (0–12)	2.1 ± 3.8 (0–13)^$^	n.s
ARS preoperative	7.6 ± 5.1 (0–14)	6.6 ± 5.0 (0–16)^$^	n.s
KOOS subscale scores			
Symptoms	81.2 ± 22.1 (29–96)	76.4 ± 16.2 (46–100)	n.s
Pain	89.6 ± 12.3 (58–100)	80.7 ± 14.8 (44–100)	n.s
ADL	94.7 ± 6.6 (84–100)	86.8 ± 12.0 (65–100)	n.s
Sports and recreational activities	85.0 ± 15.7 (55–100)	59.3 ± 26.1 (15–100)	0.006^$^
QoL	69.3 ± 18.4 (31–100)	62.5 ± 23.0 (25–94)	n.s
Post-operative reduction in sports activity			
None, *n* (%)	5 (46%)	5 (24%)	
Yes, because of knee joint complaints, *n* (%)	4 (36%)	13 (62%)	
Yes, because of other reasons (family, career, other interests), *n* (%)	2 (18%)	3 (14%)	

Continuous variables are shown as mean ± standard deviation (range), categorical variables are shown as percentages

*Values are median; ^$^significant difference between pre- and post-operative TEGNER and ARS (*p* = 0.001); *TEGNER* Tegner Activity Scale, *ARS* Activity Rating Scale, *KOOS* Knee Injury and Osteoarthritis Outcome Score, *ADL* activities of daily living, QoL quality of life, *n.s.* not significant

In the ACL-based injury group, no statistically significant difference between pre- and post-operative Tegner Activity Scale and ARS was observed (n.s.), whereas both scores decreased significantly in the follow-up period in the PCL-based injury group (*p* = 0.001). Concerning Tegner Activity Scale and ARS, no significant difference between groups could be found pre- and post-operatively (n.s.).

At the final follow-up, all subjects with an ACL-based injury and 91% of patients in the PCL-based injury group were involved in sports activities, mostly cycling and swimming (Table 3). In general, frequency of sports activities decreased from 2.0 ± 1.5 times per week preoperatively to 1.8 ± 1.5 times at the final follow-up (n.s.) in the ACL-based injury group. In the PCL-based injury group, frequency of sports activities significantly decreased from 2.6 ± 1.3 times per week preoperatively to 1.5 ± 1.4 times post-operatively (*p* = 0.007). Group comparison (ACL-based injuries vs. PCL-based injuries) showed no statistically significant difference with respect to post-operative reduction in sports activity (n.s.) (Table 2). Concerning post-operative sports performance levels, a statistically significant difference between both groups was found (*p* = 0.045), with significant lower levels in PCL-based injuries.

**Table 3:** Details of sports activity after posterolateral corner reconstruction

Type of sport	ACL-based injury		PCL-based injury	
	Preinjury	Follow-up	Preinjury	Follow-up
Cycling	10 (91%)	10 (91%)	20 (95%)	18 (86%)
Jogging	7 (64%)	6 (55%)	13 (62%)	4 (19%)
Soccer	8 (73%)	1 (9%)	7 (33%)	2 (10%)
Skiing	2 (18%)	1 (9%)	4 (19%)	4 (19%)
Swimming	8 (73%)	8 (73%)	11 (52%)	10 (48%)
Hiking	5 (46%)	5 (46%)	10 (48%)	5 (24%)
Fitness	3 (27%)	3 (27%)	10 (48%)	8 (38%)
Volleyball	3 (27%)	2 (18%)	1 (5%)	1 (5%)
Table Tennis	3 (27%)	3 (27%)	3 (14%)	1 (5%)
Badminton	2 (18%)	1 (9%)	4 (19%)	1 (5%)
Dancing	1 (9%)	1 (9%)	3 (14%)	2 (10%)

*ACL *anterior cruciate ligament*, PCL *posterior cruciate ligament

Values are expressed as number of patients (percentage of patients) who performed the sport activities before the injury and at follow-up. Multiple answers were possible

### Knee Injury and Osteoarthritis Outcome Score (KOOS)

Post-operative KOOS subscales were generally higher in the ACL-based injuries; however, statistical significance was only reached for the KOOS subscale sports and recreational activities (85.0 ± 15.7 vs. 59.3 ± 26.1, *p* = 0.006) (Table 2).

### Return to work

The average time to return to work was significantly shorter in the ACL-based injury group as compared to the PCL-based injury group (10.9 ± 3.9 weeks vs. 21.3 ± 10.4 weeks; *p* = 0.003).

Preoperatively, 27% (*n* = 3) of the subjects in the ACL-based injury group had an occupation involving heavy physical work, while 73% (*n* = 8) worked in occupations that required little physical work. Post-operative physical workload did not change in 82% (*n* = 9), whereas improvement or deterioration was observed in 1 patient each.

In the PCL-based injury group, 62% (*n* = 13) had an occupation including heavy physical workload preoperatively, while 38% (*n* = 8) patients worked in occupations that required little physical work. Post-operatively, 67% (*n* = 14) showed no change in physical workload, whereas deterioration was observed in 24% (*n* = 5). Group comparison showed no statistically significant difference with respect to changes in workload (n.s.).

## Discussion

The most important finding of the current study was that sports activity (Tegner Activity Scale, ARS) significantly decreased post-operatively in PCL-based injuries, whereas no significant change was observed in ACL-based injuries. Furthermore, significantly more patients had to reduce the frequency of sports activities in the PCL-based injury group. Second, time to return to work was significantly longer in PCL-based injuries. Another important finding was that injury mechanisms differed significantly between ACL- and PCL-based PLC injuries. Whereas most ACL-based injuries were sports injuries, PCL-based injuries usually occurred due to traffic accidents.

While clinical outcomes after PLC-R with additional ligamentous injuries are well described [2, 4, 9, 11, 12, 17, 25, 34, 41, 43], the available literature mainly focused on the outcome regarding complications, re-instability, and functional scores. Only one clinical study by *Wajsfisz *et al*.* assessed the functional outcome and return to work following PLC-R with additional ACL-R or PCL-R [41]. However, there is a lack of evidence when comparing sports activity and especially return to work between ACL-based and PCL-based PLC injuries.

So far, research on return-to-sport activities has concentrated on results after isolated ACL-R or PCL-R [8, 14, 23, 26, 27, 46]. However, after isolated ACL-R, return-to-sport activities is mostly possible with clear limitations in terms of the post-operative sports activity level [14, 26, 27]. Return to sports after isolated PCL-R was only 44% in a systematic review by *Devitt *et al*. *[8], and restrictions to post-operative activity levels [46], sport activities, and physical performance were reported [23]. These results are further strengthened by the present study on combined ligamentous injuries which also showed that patients were rarely involved in high-impact sports post-operatively (Table 3). In terms of patient education, this may require further preoperative expectation management of young and active patients with PLC injuries and additional rupture of the anterior or posterior cruciate ligament.

It is known that additional injuries to the PLC, such as cruciate ligament tears, have a negative impact on the clinical outcome [4, 40]. It has been reported that post-operative functional gains after isolated PCL-R are comparable to isolated ACL-R. However, mean preoperative scores in PCL patients are lower and therefore end up at a lower final score compared to ACL patients [31]. Compared to a study after isolated ACL-R and PCL-R [31], KOOS subscales in the current cohort were higher for both groups even though the PLC was additionally reconstructed. In contrast to the results of *Wajsfisz *et al*.* following PLC-R with additional ACL-R or PCL-R [41], significant group differences in the KOOS sports and recreational activities subscale could be shown. Similar to their results, no significant group differences in other KOOS subscales were evident. Furthermore, KOOS subscales in our ACL-based injury group were comparable to the results of *Cartwright *et al*.* after combined PLC and ACL-R [4].

In the present study, a significant deterioration of Tegner Activity Scale, ARS, and frequency of sport activities could be demonstrated for the PCL-based injury group. Reasons for lower scores after combined PLC- and PCL-R are likely to be associated with heterogeneous aetiologies with higher exposures to high-impact injury mechanisms with following injuries to deep ligamentous structures that are not addressed sufficiently by PLC-R and PCL-R. In line with the literature, time to surgery in our cohort was longer in the PCL-based injury group [34]. This may explain the restrictions to post-operative return-to-sport activities and further existing knee joint complaints, since further progression of knee osteoarthritis due to ligamentous instability could have taken place in that prolonged time to surgical reconstruction [3].

Another important finding of the present study was that the return-to-work time was significantly prolonged in the PCL-based injury group with post-operative work load deterioration in 24% of patients. The long return-to-work time after PCL-R might be due to the rehabilitation protocol including strict non-weight bearing with immobilization in full extension and posterior tibial support, followed by functional bracing for up to 6 months [19]. Our results have strengthened the assumption that, compared to isolated ACL or PCL injuries [15, 44], a high return-to-work rate can be expected for patients after PLC-R with additional ACL-R or PCL-R. With our study, an improved patient education regarding return-to-work time and possible changes in work load following such complex ligamentous knee injuries can be achieved.

Various techniques have been described to treat acute or chronic PLC injuries. In the present study, the *Larson* technique [22] has been used to reconstruct the PLC. However, there has been a significant change in recent years towards a more anatomic technique. More recently, the *LaPrade *et al*.* [20] or the *Arciero* [1] technique is preferred at the authors’ institution. Therefore, further research after more anatomic PLC reconstruction techniques is indicated.

Along with certain strengths, there are some limitations to this study. First, this is a retrospective study. On the other hand, there are no other studies reporting on physical activity and return to work after PLC-R with concomitant ACL-R or PCL-R. Second, the number of patients in this study is low. However, this is attributable to the low incidence of the injury patterns studied. Furthermore, the statistical power of the current study has been shown to be 0.8. Third, only the PLC-R technique described by *Larson *et al*.* [22] was used in this study. Thus, concerning the PLC intervention, a more homogeneous cohort was achieved. Fourth, additional meniscus injuries were not excluded from this study. However, according to previous research, meniscus lesions did not affect return-to-sports activity after ACL-R [27].

The results of this study have clinical relevance when considering post-operative outcomes, including physical activity and work ability, after reconstruction of ACL- or PCL-based PLC injuries. In future, findings of this study can clinically help to improve preoperative patient counselling regarding post-operative expectations.

## Conclusion

High rates of return to sports and work can be expected after combined PLC-R in both ACL- and PCL-based injuries. However, deterioration of sports ability must be expected in PCL-based injuries. ACL-based injuries led to superior patient-reported outcomes and an earlier return to work, as compared to PCL-based injuries.
